# A Novel Software Architecture for the Provision of Context-Aware Semantic Transport Information

**DOI:** 10.3390/s150612299

**Published:** 2015-05-26

**Authors:** Asier Moreno, Asier Perallos, Diego López-de-Ipiña, Enrique Onieva, Itziar Salaberria, Antonio D. Masegosa

**Affiliations:** 1 Deusto Institute of Technology (DeustoTech), University of Deusto, Bilbao 48007, Spain; E-Mails: asier.moreno@deusto.es (A.M.); dipina@deusto.es (D.L.-I.); enrique.onieva@deusto.es (E.O.); itziar.salaberria@deusto.es (I.S.); ad.masegosa@deusto.es (A.D.M.); 2 IKERBASQUE, Basque Foundation for Science, Bilbao 48011, Spain

**Keywords:** Intelligent Transportation Systems, multimodal transport information, semantic middleware, Linked Open Data, context-aware computing

## Abstract

The effectiveness of Intelligent Transportation Systems depends largely on the ability to integrate information from diverse sources and the suitability of this information for the specific user. This paper describes a new approach for the management and exchange of this information, related to multimodal transportation. A novel software architecture is presented, with particular emphasis on the design of the data model and the enablement of services for information retrieval, thereby obtaining a semantic model for the representation of transport information. The publication of transport data as semantic information is established through the development of a Multimodal Transport Ontology (MTO) and the design of a distributed architecture allowing dynamic integration of transport data. The advantages afforded by the proposed system due to the use of Linked Open Data and a distributed architecture are stated, comparing it with other existing solutions. The adequacy of the information generated in regard to the specific user’s context is also addressed. Finally, a working solution of a semantic trip planner using actual transport data and running on the proposed architecture is presented, as a demonstration and validation of the system.

## 1. Introduction

Progress made over the last years in the application of ICT to transportation systems is extensive, constant and diverse. With regard to the software services for transport, some of the elements that have evolved the most, providing a high added value to the user, are the multimodal trip planning solutions. In this area, solutions like *Google Maps* or *OpenTripPlanner* have made important progresses in facilitating trip management and planning to the users.

Efforts are also being made at institutional level to provide the tools that allow citizens to opt for sustainable transport solutions. Thus, according to data from the International Association of Public Transport (UITP) [1], it is expected that public transport by 2025 will double its market share compared to 2009, thereby completing the transition to a sustainable transport model.

However, there is still much room for improvement in this area. Existing tools are not sufficiently interoperable due to the lack of a universal and consistent format to represent transport information. Likewise, they do not take into account relevant factors, such as the user’s context. Moreover, in most cases these planning tools are closed so the access to its information becomes very costly.

This paper proposes a novel software architecture to address the above-mentioned limitations by incorporating innovative technologies such as semantic middleware, context-awareness computing or Linked Open Data, given that they have already been successfully tested in other application areas.

The article is structured into four sections. Section 2 provides an overview of the state of the art related to the technologies and knowledge areas covered in the proposed solution. Section 3 details the design and implementation of the Multimodal Transport Ontology (MTO) used for the management of transit information. Section 4 establishes the design of a distributed software architecture for semantic information provision allowing dynamic integration of transport data, detailing its components and characteristics. In Section 5, the overall system is validated by the deployment of a trip planning solution running on actual transport data. Finally, conclusions and future work derived from the experimentation analysis are given.

## 2. State of the Art

The proposed architecture is based on several areas of knowledge. This section aims to show an overview of the state of the art within these areas. The main research field will be introduced first: Advanced Traveler Information Systems (ATIS). Then, existing solutions for multimodal transport data management will be evaluated, as well as successful alternatives for semantic data provision.

### 2.1. Advanced Traveler Information Systems

Transportation systems efficiency is essential for economic development. Intelligent Transportation Systems (ITS) can be defined as a set of applications within computer science, electronics and communications that, from a social, economic and environmental point of view, are aimed at improving mobility, security and transport productivity, optimizing the use of infrastructures and energy consumption and improving the capacity of the transport systems [2].

Within the ITS field, this work is focused on providing enriched transport information to the user through the integration of information sources and the generation of new knowledge. The most relevant research works in this area are focused on ATIS, designed to assist travelers in planning their trips and route optimization [3,4].

These systems use ICT in order to collect, process and distribute the latest traffic information, road conditions, travel time, expected delays, alternative routes and/or weather conditions to the user, giving travelers the opportunity to make informed decisions about when to travel, what mode of transport to use or which route to take.

Research done in these concepts has been instrumental in the development of software tools and commercial applications for journey planning, one of the areas with greater acceptance within the ATIS. Examples of it are *Google Transit* or *Moveuskadi* in the private field or *OpenTripPlanner* as open source software.

### 2.2. Transit Information Formats and Standards

Transportation companies have their own information about service planning related to routes and schedules made by their fleets of vehicles. But as Campbell *et al*. [5] indicate on their work, the effectiveness of transportation information systems depends largely on the ability to integrate information from diverse sources and the suitability of this information to the specific user.

Currently there are initiatives for the publication and exchange of carriers’ transit information, which are allowing developers to consume this information and to integrate it in their applications in an interoperable way. The two main existent solutions (as to their widespread use and community support) for transit data modelling and publishing are GTFS, from Google, and WFS from the Open Geospatial Consortium (OGC) along with several ad-hoc solutions defined by transport agencies themselves. The details of these solutions are described below.

#### 2.2.1. General Transit Feed Specification (GTFS)

The aforementioned applications use GTFS as the data format for modelling transit information. GTFS defines a common format for public transportation schedules and associated geographic information, having established itself as the *de facto* standard for the representation of transit data, thanks in large part to the support received from Google and its maps services. However, the format relies on comma-separated value (CSV) files to represent the information which is then compressed and stored. This leads to isolated and outdated data that is neither easily queryable nor extensible.

#### 2.2.2. Web Feature Service (WFS)

The Open Geospatial Consortium Web Feature Service Interface Standard (WFS) provides an interface allowing requests for geographical features across the web using platform independent calls. The XML-based GML furnishes the default payload encoding for transporting the geographic features.

The WFS specification defines interfaces for describing data manipulation operations of geographic features. Data manipulation operations include the ability to get or query features based on spatial and non-spatial constraints and create/update/delete new feature instances. Implementations of the WFS standard are however scarce, mainly due to the complexity of the data model and the verbosity of the required queries.

#### 2.2.3. Ad-Hoc Solutions

There are also other systems to represent and/or provide transit information, mostly defined by agencies or institutions that manage their own data. One of the most prominent is *TransXChange* used in the United Kingdom and Australia to interchange bus service planning information.

Therefore, various systems coexist in order to represent transit information. WFS specification is focused to make queries on geospatial data stored in Geographic Information Systems (GIS) and its use is complex. Ad hoc solutions are optimal in their domain but they are not interoperable. GTFS is the *de facto* standard, however, the treatment and consultation of its data is not trivial, because they are not structured and do not allow the inclusion of qualitative attributes of the route (like ecological or tourist interest) which are increasingly relevant when making schedules.

Table 1 shows a schematic comparison between the aforementioned formats and MTO, the proposed solution for the management and provision of multimodal transport information. A set of features, considered relevant for data integration and interoperability are presented.

**Table 1:** Comparison between formats for transport information provision.

	GTFS	WFS	Ad-Hoc Solutions	MTO
**Classification**	Open Data	Open Data	Private	Open Data
**Structure**	CSV	GML (XML)	Variable	Formal Ontology
**Extensibility**	No	No	No	Yes
**Linkable**	No	No	No	Yes
**Queryable**	Programmatic	Web Service	API	Direct (SPARQL)
**Data Access**	Complete	Limited	Variable	Complete

*Classification* defines the legal restrictions applied to the provided data (e.g., open access and redistributable or privative).

*Structure* refers to the format of the data. It is important to classify these formats into non machine-readable (e.g., PDF file) and machine-readable (CSV, XML, taxonomy, *etc.*)

*Extensibility* defines the intrinsic capability of the format to be extended with other relevant data thereby enriching the original data.

*Linkable* exposes the ability of the format to be included in other datasets as a reference (e.g., using URIs) allowing even greater enrichment.

*Queryable* and *Data Access* make reference to the accessibility of the data in relation to the consultation mechanism and the completeness of the information provided, respectively.

Thus, GTFS provides complete access to the data but has to be done programmatically, with a custom scraper, as no interface is provided. WFS provides an interface but the queries are predefined so the access is limited. MTO provides complete access through standard yet customizable SPARQL queries allowing the user to get the information needed, even enriched using the mechanisms provided to link the data with other relevant datasets. Section 4 extends this description with a more detailed view of the system architecture including some example queries and SPARQL specification details.

### 2.3. New Trends in Transit Information

**Figure 1:**
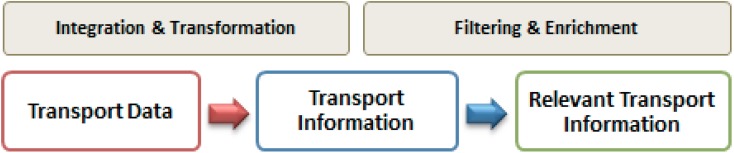
Evolution of transport information.

One of the main objectives of the proposed architecture is to improve the relevance of the transport information currently provided to the user. In the last years, the efforts on user services and transportation information have been focused on data interoperability in order to obtain this value added information. Figure 1 shows the evolution of the transport information and its transformation through different processes making it more relevant for the user.

In order to support these processes and provide solutions to new (quantitative and qualitative) information integration, sharing and aggregation limitations, there are studies about the possibilities offered by relatively recent emerging paradigms in the computing field, such as ontologies and the Web of Data. We describe some of these studies in the next subsections.

#### 2.3.1. Ontologies

The work carried out by the Artificial Intelligence community showed evidence that formal ontologies could be used to specify knowledge between different entities [6,7].

In computing, the ontology term refers to the formulation of a comprehensive and rigorous conceptual schema given within one or more domains [8]. Being independent of language and understandable by computers, ontologies are useful because they help to achieve a common and integrated understanding of descriptive information. This not only makes it easier for other human users to understand the specifically intended meaning of the models, but also means that other tools can use the definitions transparently [9].

#### 2.3.2. Semantic Web

Ontologies are the heart of the Semantic Web, an extension of the World Wide Web in which the meanings (semantics) of information and services are defined [10] allowing to satisfy the requests of people and machines using web content. On the web there are millions of resource accesses, which have brought a lot of success, but also provoke problems of information overload and sources heterogeneity. The Semantic Web helps to solve these problems reducing the cognitive effort of users delegating them to agents that can reason, and make inferences to offer accurate information.

To achieve this goal of classification and provision of relevant information, Semantic Web applications used a set of standardized languages and technologies (essentially RDF, SPARQL and OWL) collaborating between them to turn the web into a global infrastructure where data and documents can be shared and reused.

#### 2.3.3. Linked Data

Once established the formats (RDF, OWL) used to model specific vocabularies, thus generating ontologies for specific domains, and having defined the semantic web as an infrastructure to support these domains, the next step is to integrate this vocabularies or, in other words, link the data. The concept of Linked Open Data (LOD) arises so as the mechanism to interconnect RDF datasets available on the Internet using the HTTP protocol, as with HTML documents.

In computing, Linked Data describe a method to publish structured information, so that it can be interconnected and thus be more useful. It is based on standard web technologies such as HTTP, RDF and URIs, but instead of using them to serve web pages to people, it extends them to share information in a way that can be processed automatically by computer. As the resulting Web of Data is based on standards and a common data model, it becomes possible to implement generic applications that operate over the complete data space. This allows connecting and retrieving relevant data from various sources [11].

In order to standardize and measure the quality of the published datasets which are beginning to be huge, in 2010 a metric which is known as “*5-star Linked Data*” was introduced by Berners-Lee [12]. Basically, it consists on a set of incremental characteristics that should meet the published data to be considered as Linked Data under community criteria. The more functionality the dataset meets, the better the quality of it. Table 2 shows that classification.

**Table 2:** *5-star* Linked Data rating system.

Stars	Description	Acronym	Example
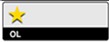	Available on the web	OL: On-Line	PDF
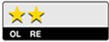	Available as machine-readable structured data	RE: Readable	XLS
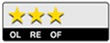	Non-proprietary format	OF: Open Format	CSV
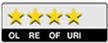	Using URIs to denote things	URI: Universal Resource Identifier	RDF
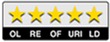	Link data to related datasets	LD: Linked Data	RDF

This concept has gained momentum after the publication of large semantic resources as DBpedia or Bio2RDF and the announcement by some governments about its decision to make public their data on a set of Open Government initiatives.

In October 2007 datasets of more than two billion triples (a data entity used for storing semantic information composed of subject-predicate-object) were counted, interlinked by means of more than two million RDF links [13]. In September 2011 this information had grown to thirty-one billion RDF triples and five hundred million links. Such efforts, both institutional and personal, are resulting in the publication of data under the characteristics and infrastructure of the Semantic Web.

### 2.4. Integrating LOD for Multimodal Transportation

Capabilities to promote knowledge sharing, structuring such knowledge and interoperability between systems have favored the use of ontologies and LOD in many areas of study and different application domains like medicine [14,15], education [16,17] or logistics [18,19]. Therefore, it should not be surprising to find similar work in the field of transportation information, motivated by various objectives, methods and expected results, but all under the premise of semantic data modeling.

#### 2.4.1. Geospatial Data Management

Interoperability is becoming essential for geographic information systems whose information is usually stored in geospatial databases, accessible only via GIS. However, these sources are increasingly heterogeneous so it is necessary to consider this heterogeneity and favour methods that enable interoperability between geographic tools in order to satisfy the growing demand in the use and sharing of geospatial data [20,21].

Traditional GIS systems perform spatial queries using a method based on keywords. This approach is unable to fully express the user’s needs, because of the lack of geographic concepts (semantics) in the data set. In this context, the most promising approach to end this ambiguity is the implementation of geospatial semantics using ontologies for geographic datasets.

Lorenz *et al.* made a comprehensive survey analysis in their work Ontology of Transportation Networks [22], where points out the efforts made by international institutions in order to standardize geographical information and explained the ontology of a transportation network derived from the ISO Geographic Data Files.

The thesis work presented by Lemmens [23] delves into the need to seek interoperability between different datasets and web services based on geographic information. It exposes how the paradigm change in terms of software architecture (from a centralized mode to a distributed and interconnected one) has influenced substantially in the available geographical information tools.

On the other hand, Zhao [24] presented a paper where geographical data interoperability is tackled from a distinct perspective. While the authors cited above focus their efforts on geographic information semantic modeling, designing one or more ontologies to try to get a direct translation to a richer semantic model for geospatial formats, Zhao seeks to reuse existing standard formats and geospatial protocols and enhance them using semantics in the query layer.

He argues that the direct conversion of all present geospatial data (stored mainly in geographical databases) to an ontological model is not a viable alternative because the process would be prolonged in time, being subject to the occurrence of errors and inefficient. Furthermore, existing tools for ontology management (such as Protégé) would not be able to bear such a heavy burden of instances mainly due to the required memory consumption.

#### 2.4.2. Transport Information Modelling

Although several authors have tried to manage GIS systems’ geographical information by ontologies, it has been proven that the complexity of this type of information, as well as its capability requirements make it impractical to compute large-scale application of such solutions. Therefore, the current approach is to direct the research towards domains or more specific areas within the transport information.

Niaraki [25] developed an ontology-based personalized route planning system using multi-criteria decision making. Another related work by Houda [26] focused on the information required by the passenger for preparing a journey, choosing the best way to move from one point to another using multimodal transportation. A work by Gunay [27] presents a more general solution, generating a semantic geoportal based on the Infrastructure for Spatial Information in the European Community (INSPIRE) data theme. The aim of this work is to investigate the use of semantics to empower the traditional GIS approach.

Along with the use of ontologies, the inclusion of contextual information also derives in the enrichment of the resulting information [28]. Examples of these are the recent solutions presented by Kim [29] or Bujan [30] for context management in the fields of healthcare and tourism, respectively.

All the research discussed above concerns ontology studies. However, each work is motivated by different objectives, methodologies or expected results. Our goal is to construct a distributed software architecture that allows, through the formalization of transit data acquired from heterogeneous sources together with the integration of relevant information, the creation of software services related to multimodal mobility.

## 3. MTO: Multimodal Transport Ontology

Section 2 highlighted the limitations encountered with the definition of a language that allow, on the one hand, to formally model transport information and, on the other, to facilitate its consultation and provision to the user. Since these features are very satisfactorily resolved through the use of ontologies and Linked Open Data as demonstrated in other areas, the establishment of an ontology as a format for the management and provision of multimodal transport information is conducted.

### 3.1. Design Methodology

A body of formally represented knowledge is based on a conceptualization: the objects, concepts, and other entities that are assumed to exist in some areas of interest and the relationships that hold among them. A conceptualization is an abstract and simplified view of the world that we wish to represent for some purpose. An ontology is an explicit specification of this conceptualization and the design of ontologies should follow a process to guide and evaluate this model. Several studies can be found, an example is the criteria identified by Gruber [6] for ontologies whose purpose is knowledge sharing and interoperation among programs based on a shared conceptualization.

There are also more detailed methodologies that seek to establish a strategy for the guided development of ontologies. In 1995 Uschold and King [31] suggested a methodology based on the following phases: identify the target ontology, build, evaluate, and document it. A more ambitious approach is the so called Methontology [32] which covers the whole life cycle, matching the ontology to a software product. Noy and McGuiness [33] for their part, describe an iterative process consisting of several stages: determine the domain and scope, reuse existing ontologies, list the important terms, define classes and their hierarchy, define properties and constraints and create the instances.

In 2010, Suarez-Figueroa [34] presented a new approach with NeOn methodology. This approach for building ontology networks is a scenario-based methodology that supports the collaborative aspects of ontology development and reuse, as well as the dynamic evolution of ontology networks in distributed environments. NeOn exposes a set of nine scenarios for building ontologies and ontology networks, emphasizing the reuse of ontological and non-ontological resources, reengineering and fusion, and considering collaboration and dynamism.

NeOn Methodology is the most suitable approach for the design of MTO, primarily due to the fact that is focused on the reuse and transformation of non-ontological resources, such as the CSV files that compose GTFS. The development is mainly ascribed to the second scenario, using adapters which allow the generation of semantic information from data sources currently available on the Internet.

### 3.2. GTFS Adapter

Given that GTFS is currently the de facto standard for transit data representation, it has been chosen to undertake a reformulation of their CSV files to entities within the ontology. A survey of existing transportation and geographical vocabularies that could be reused for the ontology design has been conducted. Thus, widely community supported vocabularies, like GeoNames [35], GeoSPARQL [36], Time [37] or WGS84 [38] have been used.

Also, an adapter, a desktop portable and multiplatform application implemented in Java, has been developed for the conversion from GTFS to MTO. Its functionality and technical characteristics are:

1. Selection and validation of GTFS files compressed in ZIP format. Extraction of the CSV files contained in the ZIP corresponding to the different concepts of a transit operation.
2. Loading of the MTO OWL file with the ontological base model. This model has been developed via OWL API, a Java API and reference implementation for creating, manipulating and serializing ontologies.
3. Transformation of non-ontological resources: establishment of entities, properties and constraints generating unique indexes for each transit agency and conversion of specific individuals from CSV files.
4. Generation of semantic appropriate links and hierarchy including links to external vocabularies like GeoNames and creation of the instances.
5. Serialization of OWL resulting files (to facilitate its treatment the number of files depends on the size of the original GTFS file).

### 3.3. MTO Specification

The pursued goal with the definition of an ontological data model is to integrate and structure existing multimodal transportation data, obtaining a semantic model for the representation of transport information. This model will have the ability to extend and link related relevant information and will be supported with tools that enable the effective consultation of its information.

As specified previously, MTO has been developed as an OWL2 ontology file, a semantic extension over RDF (XML standard for resource description) generated in 2009 by the W3C. Thanks to the use of ontologies to define the transport data model, the knowledge can be easily processed and shared between the components of the system. It also favored interoperability with other transport systems.

To facilitate the management of the ontology, it is published as two separate OWL files. Both files are needed for the operation of the ontology, so the distribution of them as a bundle is compulsory:

- *mto-core.owl* with the ontological base model; establishing the entities, properties and constraints of the ontology but without links to other vocabularies nor individuals or concrete instances.
- *mto-top.owl* with all the references generated for the entities and properties imported from other vocabularies and linked to the base ontology. *mto-core.owl* imported this file.

**Figure 2:**
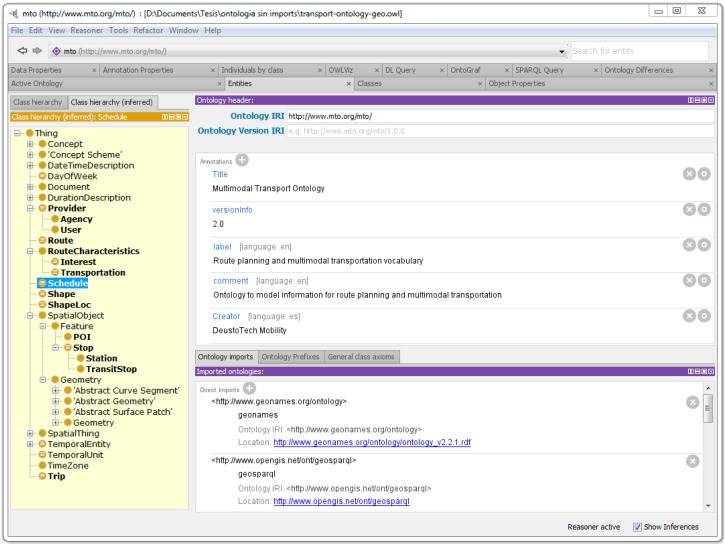
MTO OWL file in Protégé.

Protégé, a free, open-source ontology editor and framework for building intelligent systems has been used for the development of MTO. Figure 2 shows the ontology loaded in the editor.

The ontology description can be seen in the foreground, with the title, IRI and version of the ontology, among other information. On the bottom of the UI the imported vocabularies are shown; in this case GeoNames and GeoSPARQL. The left panel in yellow shows the ontology class hierarchy, with the entities corresponding to MTO highlighted in bold. The MTO vocabulary as well as the external vocabularies imported for the generation of the transport data model will be explained in more detail in the following subsections.

#### 3.3.1. MTO Vocabulary

**Figure 3:**
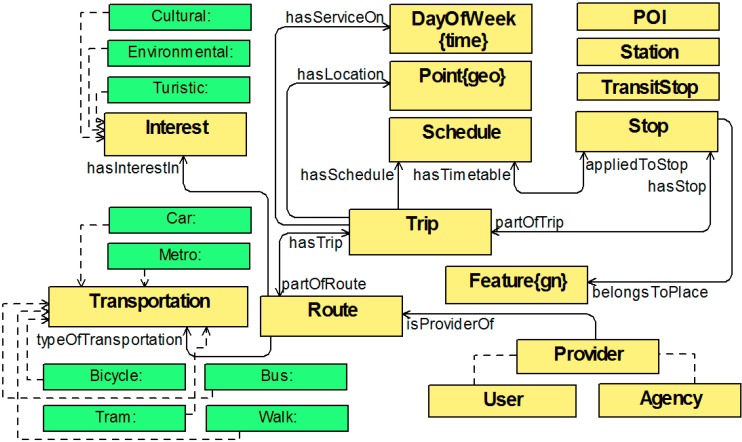
Multimodal Transport Ontology main concepts and relationships.

As indicated above, the basis for the transport data model will be taken from GTFS specification. The main classes, properties and relationships of the MTO vocabulary along with a functional description of them and its correspondence in GTFS are explained below and shown in Figure 3.

- *Provider*. Abstract class that will act as a parent class for all data providers. Has no equivalence in GTFS.
- *Agency*. One or more transit agencies that provide the data. Inherits from *Provider*. Equivalent to agency.txt file.
- *Route*. Transit routes. A route is a group of *Trips* that are displayed to riders as a single service. Equivalent to routes.txt file.
- *RouteCharacteristics*. Abstract class that will act as a container for the different route options defined. Has no equivalence in GTFS.
- *Transportation*. Means of transport used on a *Route*. Inherits from *RouteCharacteristics*. Has no equivalence in GTFS.
- *Trip*. Trips for each *Route*. A trip is a sequence of two or more *Stops* that occurs at specific time. Equivalent to trips.txt file.
- *Stop*. Individual locations where vehicles pick up or drop off passengers. Inherits from *Feature* (GeoSPARQL). Two significant properties: *hasGeometry* establishing a standard format to define the geometry of the particular point, and *belongsToPlace* (GeoNames) linking point position with the related geopolitical entity. Equivalent to stops.txt file.
- *TransitStop*. Specialization of *Stop* class for stops that are part of a *Trip*. Inherits from *Stop*. Has no equivalence in GTFS.
- *Schedule*. Times when a vehicle arrives at and departs from individual *Stops* for each *Trip*. Equivalent to stop_times.txt file.
- *Shape* & *ShapeLoc*. Rules for drawing lines on a map to represent a transit organization's routes. Equivalent to shapes.txt file.
- *POI*.* Extension of the transport base ontology. Define tourist sights. Inherits from *Feature* (GeoSPARQL). Has no equivalence in GTFS.
- *POIClassification*.* Extension of the transport base ontology. Define a classification for the *POIs*. Has no equivalence in GTFS.

#### 3.3.2. Geographic Information Management

One of the key characteristics for a system which aims to model transportation information is the design used to store the geographical data. In this aspect, the decisions that have been taken are based on the standardization and future extensibility and/or reusability of the designed ontology.

**Figure 4:**
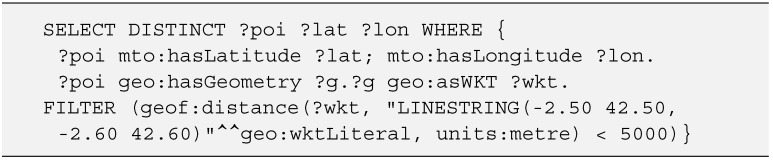
SPARQL query. Selecting POIs within 5 km of a given line.

Regarding standardization this work has followed a twofold approach: geo-referenced points have been defined by using the widely supported properties latitude and longitude, following the WGS84 standard. *hasGeometry* property has also been defined, extending from the GeoSPARQL ontology and allowing to perform geometric queries over ontology points, as shown in Figure 4.

As can be seen in the code snippet, the query performs a selection of POIs, including its position. To filter the resulting data, the function *geof:distance* is being used with relation to a geometric line. These kind of geometric filters are provided by GeoSPARQL, an OGC standard for geospatial semantic data. For these functions to be operational, they must be supported by the SPARQL endpoint.

**Figure 5:**
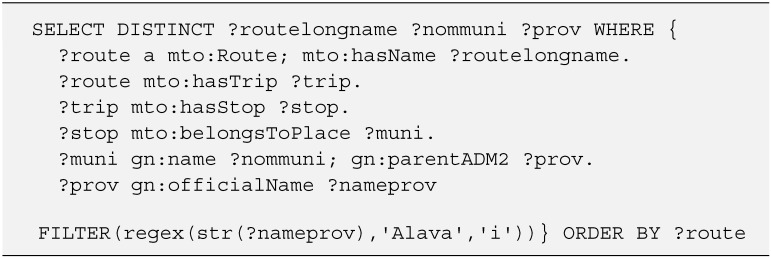
SPARQL query. Selection of routes located in a particular province/jurisdiction.

It has also been decided to link the ontology with GeoNames, an ontology engaged in storing geopolitical information. The link is done through the *belongsToPlace* property referencing the GeoNames URI corresponding to the territory where the point of the ontology is located. Figure 5 shows an example query using this link with GeoNames ontology.

In this case the query performs a selection of transit routes, including its name and the route location. To filter the resulting data, geopolitical information about the jurisdiction in which the route takes place has been used. The property *mto:belongsToPlace* is responsible for gather this information by linking the ontology with GeoNames data.

### 3.4. Linked Vocabularies

The representation of the transport-related information as an ontology facilitates the use of advantageous aspects, like the ability to link data with other data sources, which can be relevant to the specific domain.

The enrichment phase (Figure 1) complies with the goal of adding value to the already integrated transport data through the use of collaborative information, relying on the ability of the architecture and the proposed ontology to link these data. This supposes an improvement of the information by integrating in it non-quantitative aspects.

To do so, the ontology provides the POI class, a container for collaborative points of interest that inherits from *Feature* (GeoSPARQL) to get the geographical position (latitude and longitude) of the tourist sights. It also stores the original URI of the element, its name and description, the provider of the information and the classification assigned to the POI that has been generated according to their function: services, entertainment, tourism, *etc.*

**Figure 6:**
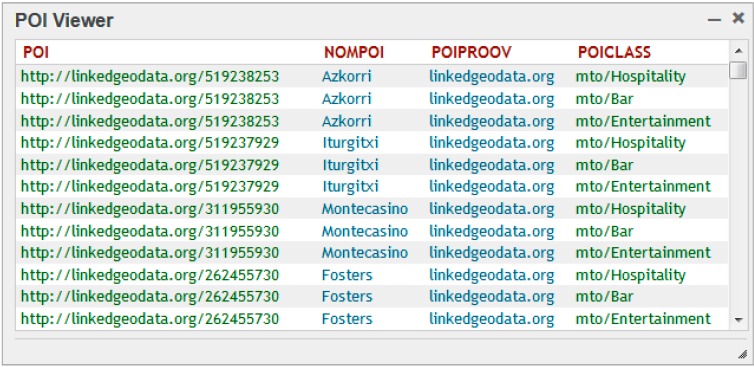
SPARQL query results. Collaborative information about restaurants in Biscay.

It was decided to extend MTO by linking and classifying the collaborative points of interest provided by LinkedGeoData and GeoNames ontologies. LinkedGeoData uses the data collected by the OpenStreetMap project and makes it available as an RDF knowledge base according to the Linked Data principles. Figure 6 shows the results of a SPARQL query over MTO asking for the LinkedGeoData [39] collaborative POIs (related to restaurants) in the surroundings of Biscay, Spain.

## 4. System Architecture

The way in which the transportation information is represented and structured supposes an innovation provided by the present work. However, the data model has to be complemented with an architecture that supports it. This is conducted by several distributed SPARQL servers. Each one of the servers maintains its own transit information and is managed in a local way, but facilitates the interoperability by means of its connection with the remaining servers by URIs, which are defined as metadata inside the ontology, allowing so, to perform distributed queries in a transparent way.

### 4.1. Distributed Transport Information

**Figure 7:**
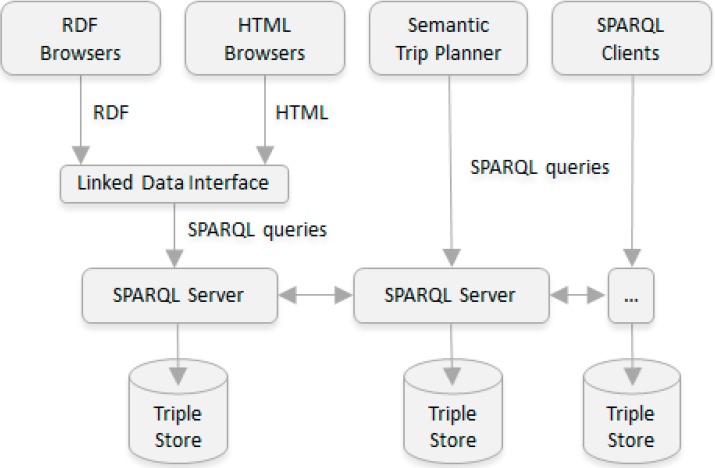
System Architecture for Multimodal Transport Information provision.

The architecture, shown in Figure 7, is similar to the one used by DNS to solve web domain names, facilitating the information distribution in a straightforward way.

When a query reaches one of the SPARQL servers, it distributes the request to the other servers hierarchically. In this case, working with geospatial information, a geopolitical classification using administrative regions (continent, state, region, town, *etc.*) has been conducted. A SPARQL server will therefore contain information about their subsidiary administrative regions thereby achieving a complete distributed model.

The main components of the proposed distributed architecture are described below:

- *Transport Information Clients*. Different types of clients (RDF browsers, HTML browsers, SPARQL clients, *etc.*) that can request the transport information provided by the system, including the semantic trip planner developed in order to validate the architecture (further described in Section 5).
- *Linked Data Interface*. Linked Data frontend for SPARQL endpoints deployed with the aim of realizing content negotiation. It provides a data interface to RDF browsers and a simple HTML interface for HTML browsers.
- *SPARQL Servers*. Distributed set of interoperable SPARQL servers. High-performance SPARQL endpoints compatible with the GeoSPARQL standard have been used, providing so, an index for geospatial queries, making it so highly indicated in the transportation domain.
- *Triple Store.* Purpose-built database for the storage and retrieval of triples through semantic queries. A triple is a data entity composed of subject-predicate-object. Each triple store maintains its own transit information as well as links to its subsidiary SPARQL servers.

### 4.2. Software Architecture

In the following points, the specific features and the criteria in the selection of the software tools for the development of the proposed architecture will be exposed.

#### 4.2.1. Linked Data Interface: *Pubby*

**Figure 8:**
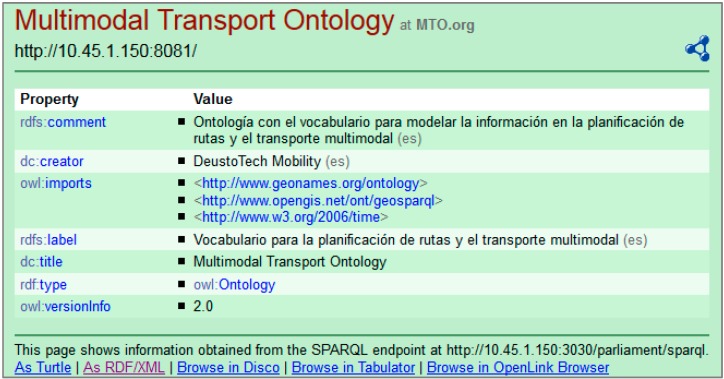
Deployment of *Pubby* for MTO information provision.

*Pubby* is an RDF server that adds a Linked Data interface to existing SPARQL-capable triple stores. Many triple stores and other SPARQL endpoints can be accessed only by SPARQL client applications that use the SPARQL protocol and cannot be accessed by the growing variety of Linked Data clients. One of the goals pursued by the proposed architecture is to enable the distribution and sharing of multimodal transport data. This objective would not be properly satisfied if the information were only accesible via SPARQL queries or using semantic applications. Figure 8 shows the deployment of *Pubby* for MTO information provision.

In RDF, resources are identified by URIs. *Pubby* will handle requests to the mapped URIs by connecting to the SPARQL endpoint, asking it for information about the original URI, and passing back the results to the client. It also handles various details of the HTTP interaction and content negotiation between HTML, RDF/XML and Turtle descriptions of the same resource.

#### 4.2.2. SPARQL Server: *Parliament*

*Parliament* is a high-performance triple store designed for the Semantic Web and compatible with the RDF, RDFS, OWL, SPARQL, and GeoSPARQL standards. It was released as an open source project under the BSD license in June, 2009. It offers a number of interesting features highly indicated for the transportation scenario:

- Employs an innovative data storage scheme that interweaves the data with a unique index. Because of that, it can answer queries efficiently by reordering query execution so that the most restrictive parts are executed first [40]. This is an important feature for the proposed solution due to the execution of complex queries related to geospatial and contextual data.
- Has a temporal index, so that it can efficiently answer queries related to time intervals.
- Supports GeoSPARQL, the newly adopted OGC standard for geospatial semantic data. Using its geospatial index, it can efficiently answer queries like “find items located within region X”.
- Includes a high-performance rule engine. This enables *Parliament* to automatically and transparently infer additional facts and relationships in the data to enrich query results. It implements RDFS inference plus selected elements of OWL (equivalent classes and properties, and inverse, symmetric, functional, inverse functional and transitive properties).

The architecture and characteristics described, as well as the support for SPARQL 1.1 Service description and Federated queries, made *Parliament* an optimal option for the storage and management of the transportation data that has to be integrated and shared by the platform.

### 4.3. Federated SPARQL Queries

SPARQL can be used to express queries across diverse data sources, whether the data is stored natively as RDF or viewed as RDF via middleware. The Federated Query specification [41] defines the syntax and semantics for executing queries distributed over different SPARQL endpoints. The SERVICE keyword extends SPARQL 1.1 to support queries that merge data distributed across the Web.

The developed architecture for semantic transport information provision makes extensive use of this specification to support the aggregation of distributed information related to multimodal mobility.

**Figure 9:**
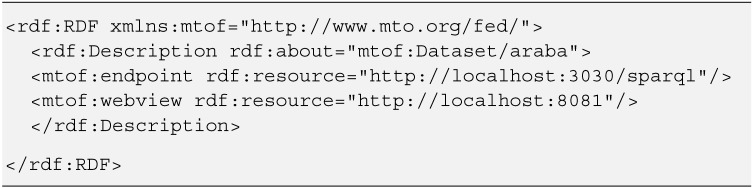
Dataset resource description, including the SPARQL endpoint URL.

To do so, a set of resources (*Datasets*) will be described in the ontology as references to other hierarchically classified SPARQL servers. In the following code fragment (Figure 9) the description of a *Dataset* for the *Araba* region, including the SPARQL endpoint URL, is established.

Once the references have been set, the queries to the server will be distributed accordingly, and the information related to one specific area will be gathered in a distributed and transparent way.

**Figure 10:**
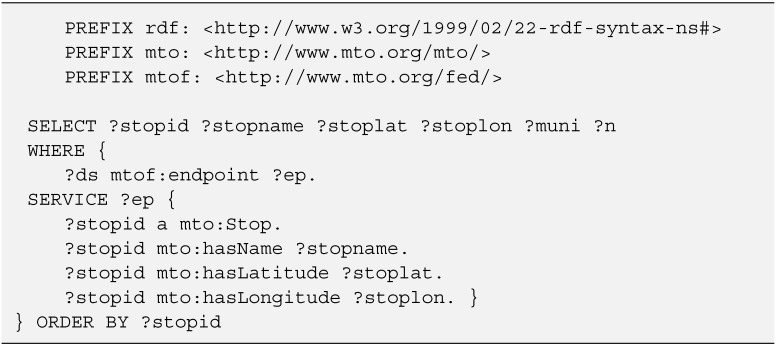
Federated SPARQL query.

Figure 10 shows an example of a federated query that receives all the information about *Stops* contained in the associated SPARQL servers. This is done using the variable *?ep* that represents a *mtof:endpoint* like the one described in Figure 9. The server iterates over all the endpoints and, using the SERVICE protocol, performs remote queries to all of them, aggregating their data and returning the information to the original queried server.

## 5. STP: Semantic Trip Planner

As pointed out in the state of the art, multimodal trip planners are one of the software tools that have advanced more in recent years within the field of ITS, providing to the user information about itineraries, timetables, routing and other transit data for an intermodal passenger transport journey.

The implementation of a semantic trip planner, an extension over the traditional journey planner, based on the integration of Linked Open Data together with collaborative information and running on the proposed architecture was established in order to validate the developed solution. The proposed Sematic Trip Planner will make use of the distributed architecture and the ontology-based data model to give users relevant information related to multimodal transportation in a more convenient way.

For the implementation of STP and the later tests we used a 64-bit machine with a quad-core 2.66 GHz processor and 8 GB of RAM. The machine was running Windows 7 and Java 7 64-bits. To deploy all the components of the architecture (Parliament, Pubby and STP) we recommend a machine with at least 4 GB of RAM with no restrictions regarding platform or OS.

### 5.1. OpenTripPlanner

The goal of the proposed solution is to provide semanticized transport information to the user in a transparent way. A multimodal trip planner with a map-based web interface is thus a good platform for testing the achievement of this goal. OpenTripPlanner (OTP), an open source platform for multimodal journey planning has been selected as a base platform for the STP software solution development.

**Figure 11:**
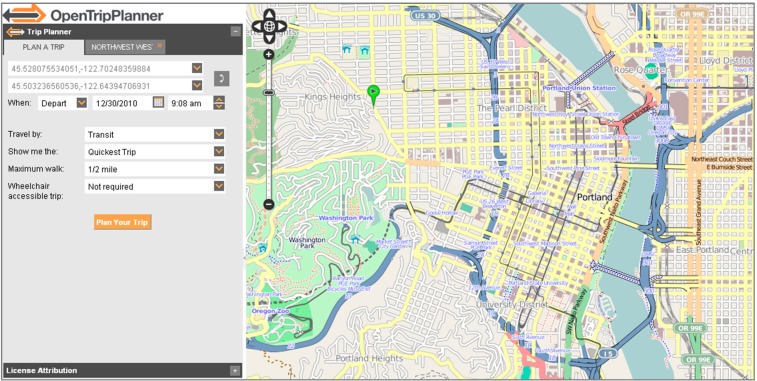
OpenTripPlanner map-based web interface.

OTP follows a client-server model, providing several map-based web interfaces (Figure 11) as well as a REST API to be used by third-party applications. It relies on open data standards including GTFS for transit and OpenStreetMap (OSM) for street networks.

Launched in 2009, the project has attracted a thriving community of users and developers, receiving support from public agencies, startups, and transportation consultancies alike. There are multiple OTP deployments and is also the routing engine behind several popular smartphone applications.

The solution presented will use the features provided by OTP, such as the map-based web interface and the routing engine. This in turn leads to an extensive modification of the OTP project (possible thanks to its collaborative and open source nature) to take, for example, the available SPARQL servers as a data source for the transport information, instead of the GTFS files.

### 5.2. STP Functionality and Improvements

Besides the deployment and initial configuration of the OTP project, which is outside the scope of this article, the following actions have been undertaken for the implementation of STP:

1. Load of transport data published by the Basque Government and generation of the corresponding semantic content via the developed GTFS adapter (detailed in Section 3.2).
2. Implementation of three distributed SPARQL servers (detailed in Section 4.1), publishing as LOD semanticized transit information relating to the three Basque country provinces.
3. OTP project modifications to include data provided by the supplied architecture:
  ○ Consuming the ontological model developed
  ○ Accessing existing related information
  ○ Providing contextualized transit information to the user

As can be seen, the last point includes several changes over OTP related with the semantic nature of the transport information now provided. These modifications will be properly described in the next subsections.

#### 5.2.1. Data Sources Configuration

OTP works with a structure called *Graph* that contains all the information about the topology of the area in which the routing algorithm has to operate. This *Graph* is generated according to the transit data provided to the system. To provide this data, an xml file has to be submitted with a property pointing to the path with the GTFS files the system will consume.

This operative has some flaws that have been satisfactorily resolved with the use of distributed SPARQL servers for storing the transit information. On the one hand, the GTFS files must be downloaded manually from each of the transit information providers (agencies, administrations, *etc.*). It should also be noted that such information must be regularly updated to maintain its accuracy.

With the deployment of STP, the xml file only stores an URL to a SPARQL server that, as mentioned previously, contains a hierarchical classification with the rest of servers. Each server maintains information about local agencies, so the update of its contents will be easier, faster and can be done via a SPARQL update query. So there is no need of storing or downloading transit data files, and the information will always remain updated.

Some changes have been made in OTP to support this operative. In addition to the configuration files, a new class inheriting from *GraphBuilder* has been created. This class controls how the transit information is processed for the building of the final topology graph. A solution for the transformation of RDF data (coming from the SPARQL servers) to CSV data, which is needed by OTP, has been implemented.

Thanks to the flexibility of SPARQL and *Parliament*, a set of standard queries have been designed that, starting from the distributed semantic information contained on the triple stores, generate CSV formatted responses with all the needed information, directly and transparently. The code snippet shown in Figure 10 is an example of this type of query, in this case receiving data about the stops on route and formatting the output in CSV.

#### 5.2.2. Context Management and LOD

An important topic to consider when generating more accurate and rich information is the specific context of the user. Without this context, data offered could lose interest, since no personalized information can be generated. For each query that arrives to STP, the user context is established with the purpose of filtering data according to their specific circumstances. The architecture defines, in order to build such model, a faceted search with a set of properties referred to the POIs in the route:

- Name of the point of interest to look for.
- Linked Data source to consult (GeoNames and/or LinkedGeoData).
- Geographical location of the query. This information can be used:
  ○ In a geopolitical way (e.g., POIs located in Biscay).
  ○ In a geometric way (e.g., POIs within 5 km of a given route).
- Hierarchical classification of the POIs
  ○ Services: Transport, Accommodation, Utilities
  ○ Entertainment: Facilities, Stores, Hospitality
  ○ Point of Interest: Cultural, Environmental, Touristic

An SPARQL query is dynamically generated based on the selections made by the user. The query can combine all of the parameters listed previously, like the geographical position of the user, the name of the POI or its classification, the proximity to the optimal route, *etc.*

**Figure 12:**
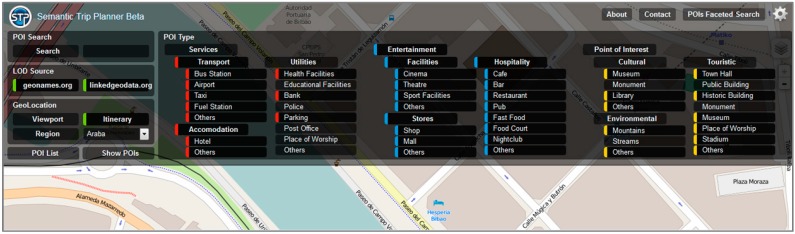
STP. Faceted search for the selection of POIs.

The query is generated transparently to the user, who simply makes use of the different options provided for that purpose in the web interface (Figure 12). Internally, the tool uses the geographic data management capabilities and the integration of relevant information to adequately answer the requests.

The generated query, resulting from the selection made by the user, could include related information coming from data sources or services that have been integrated in the ontology. In this way, the use of context and LOD can tailor information to specific user needs, customizing and improving the information given and providing relevance to it.

**Figure 13:**
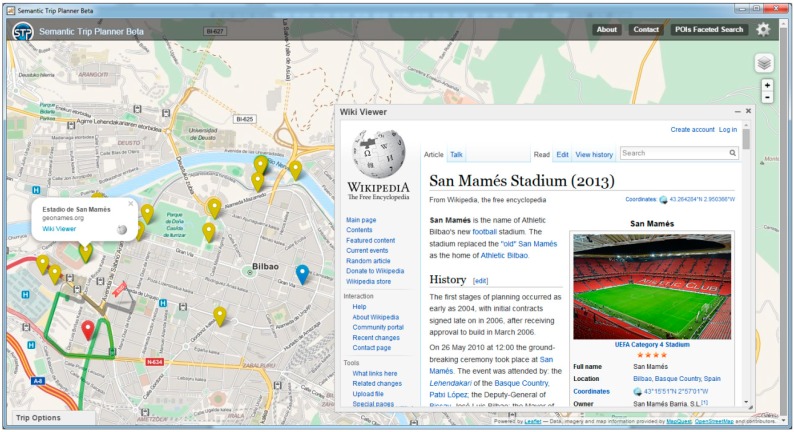
STP. Extended information (Wikipedia) about POIs.

Figure 13 shows, for example, information from Wikipedia associated with one of the POIs. This is due to the link between MTO and GeoNames, the ontology that provides such information.

## 6. Conclusions

The study conducted on the systems currently used to represent transport information led us to address the challenge of the management and representation of transport information, often based on the use of heterogeneous databases or closed non-interoperable formats that were not sufficiently interoperable, extensible or open. However, it has also shown how new trends in the management of transit data, as the generation of ontologies for representing geospatial data or the use of the Web of Data to link standard geographic information, applied to the transportation domain, can help in the task of structuring, distributing and sharing currently available information.

The work highlighted in this paper presents an alternative solution, a new approach to the management and exchange of transit data with the development of a novel software architecture, with particular emphasis on the design of the data model and the enablement of services for information retrieval, thereby providing semantic transit information, formally structured and integrated. With that, and following the strategy route pointed out by the European Commission white paper on transport [42], it is intended to improve and extend the public institutions and companies’ capability to obtain, share, and provide transport information in an integrated and sustainable way.

The publication of transport data as semantic information is established through the development of an ontology for multimodal transportation (MTO) and the design of an architecture of distributed and interoperable SPARQL servers. A much more powerful and direct tool for accessing to all the available transit data, with support for advanced queries (geospatial and geopolitical), multiple output formats and enabling dynamic integration of information.

The work done, based on the application of the Linked Open Data principles to the field of transportation has tried to improve the quality of the provided information. This promotes the development of applications that could provide innovative services in an area, sustainable mobility, which are receiving social and institutional support given its environmentally friendly characteristics. The Linked Data community is very active and hence the next steps point to an extension of the ontology and the desirable appearance of an ecosystem of both applications and open transit data that supports it.
